# Impairment of cognitive function by chemotherapy: association with the disruption of phase-locking and synchronization in anterior cingulate cortex

**DOI:** 10.1186/s13041-015-0125-y

**Published:** 2015-05-23

**Authors:** Li Mu, Jun Wang, Bing Cao, Beth Jelfs, Rosa H. M. Chan, Xiaoxiang Xu, Mahadi Hasan, Xu Zhang, Ying Li

**Affiliations:** Department of Biomedical Sciences, City University of Hong Kong, Tat Chee Avenue, Kowloon, Hong Kong; Centre for Biosystems, Neuroscience, and Nanotechnology, City University of Hong Kong, Kowloon, Hong Kong; Department of Electronic Engineering, City University of Hong Kong, Kowloon, Hong Kong; Shenzhen Key Lab of Neuropsychiatric Modulation, CAS Center for Excellence in Brain Science, Shenzhen Institutes of Advanced Technology, Chinese Academy of Sciences, Shenzhen, 518055 China

**Keywords:** Cisplatin, Decision-making, Learning and memory, Synaptic plasticity, Theta oscillation

## Abstract

**Background:**

Patients following prolonged cancer chemotherapy are at high risk of emotional and cognitive deficits. Research indicates that the brain neuronal temporal coding and synaptic long-term potentiation (LTP) are critical in memory and perception. We studied the effects of cisplatin on induction of LTP in the basolateral amygdala (BLA)-anterior cingulate cortex (ACC) pathway, characterized the coordination of spike timing with local theta oscillation, and identified synchrony in the BLA-ACC network integrity.

**Results:**

In the study presented, the impacts of cisplatin on emotional and cognitive functions were investigated by elevated plus-maze test, Morris water maze test, and rat Iowa gambling task (RGT). Electrophysiological recordings were conducted to study long-term potentiation. Simultaneous recordings from multi-electrodes were performed to characterize the neural spike firing and ongoing theta oscillation of local field potential (LFP), and to clarify the synchronization of large scale of theta oscillation in the BLA-ACC pathway. Cisplatin-treated rats demonstrated anxiety- like behavior, exhibited impaired spatial reference memory. RGT showed decrease of the percentage of good decision-makers, and increase in the percentage of maladaptive behavior (delay-good decision-makers plus poor decision-makers). Cisplatin suppressed the LTP, and disrupted the phase-locking of ACC single neural firings to the ongoing theta oscillation; further, cisplatin interrupted the synchrony in the BLA-ACC pathway.

**Conclusions:**

We provide the first direct evidence that the cisplatin interrupts theta-frequency phase-locking of ACC neurons. The block of LTP and disruption of synchronized theta oscillations in the BLA-ACC pathway are associated with emotional and cognitive deficits in rats, following cancer chemotherapy.

## Introduction

The ‘chemobrain’ refers to a range of cognitive deficits caused by prolonged chemotherapy. In clinical studies, depression and cognitive impairment were found in 40 to 70 % of patients following cancer therapy [1]. Cisplatin, a platinum compound, is an anticancer drug widely used for the chemotherapy of different malignancies. It has been shown in non-human primates that cisplatin can penetrate the Blood Brain Barrier (BBB) and accumulate in the cerebrospinal fluid of the brain [2], affect several neurobiological processes [3, 4] including oxidative stress [5]. At the cellular level, chemotherapeutic agents target the central nervous system (CNS) proliferating cells as well as changing fate decisions and cellular functions of neural progenitor cells [3]. Considering that these agents are potent neurotoxins [6], they may contribute to neurobiopathlogical processes, such as apoptosis and cell death correlated with platinum-DNA binding [6, 7], which are involved in the progress of cognitive deficiency following chemotherapy [8]. At the synaptic level, activity-dependent plasticity in synaptic strength, such as long-term potentiation (LTP), is a key mechanism in shaping cortical circuits [9, 10]. Therefore, the study of the effect of cisplatin treatment on the modulation of synaptic transmission in animal model may explain its influence on cognitive function.

The anterior cingulated cortex (ACC) is a major cortical area of the limbic loop system, integrating emotion and cognition. The anatomic connections between the ACC and the amygdala have been clearly reported previously [11, 12]. Indeed, an indirect linkage in the ACC-thalamus-amygdala circuitry [13, 14] plays an important role in synchronized function between the thalamus and amygdala, and ACC [15]. However, the synaptic metaplasticity in the amygdala-ACC circuitry following prolonged chemotherapy has not been fully explored. Therefore, we sought to characterize if chemotherapy disrupts LTP in the amygdala-ACC network integrity, which is associated with mood disorder and deficits of learning and memory.

Coordinated action-potential timing across populations of neurons is necessary for induction of synaptic plasticity [16]. Recent neurobiological studies of human memory and perception share common observations of theta frequency band oscillations in the brain and coherency of the theta oscillations and action potential activity. Rutishauser *et al.* have shown that memory formation in humans is predicted by close coordination of spike timing with the local theta oscillation [17]. This suggests that synchronized oscillatory activity promotes the communication between anatomically distant, yet functionally related, structures during learning.

In previous animal research chemotherapy-related mood and cognitive deficits have not been investigated systematically [8]. Our goal in the present study was to go beyond previous animal work, by characterizing the alteration of phase-locking of neural activity to the theta oscillation. And as such allowing identification of the disruption of the amygdala-ACC network synchrony associated with impairment of cognition and behavior after chemotherapy. In order to accomplish this we performed a series of behavioral assessments in rats following chronic administration of cisplatin, in particular the rat Iowa gambling task (RGT) was used to assess decision making functions in rats [18]. To characterize synaptic plasticity *in vivo*, we recorded evoked local field potentials (LFP) and induced LTP in the basolateral amygdala (BLA)-ACC pathways. Simultaneous recording from an array of microelectrodes allows characterization of the disruption to the phase-locking of single neural spike firing with the ongoing theta oscillation of LFP in a large scale population of neurons in the ACC and identifying the desynchronized theta activity between BLA and ACC following administration of cisplatin.

## Results

### Cisplatin suppressed spontaneous exploratory activity

To examine if cisplatin-treated rats develop anxiety-like behavior we performed open field test (OFT). The OFT is often used to assess exploration in a novel environment and offer a preliminary screen for anxiety-related behavior in rats [19].

**Fig. 1:**
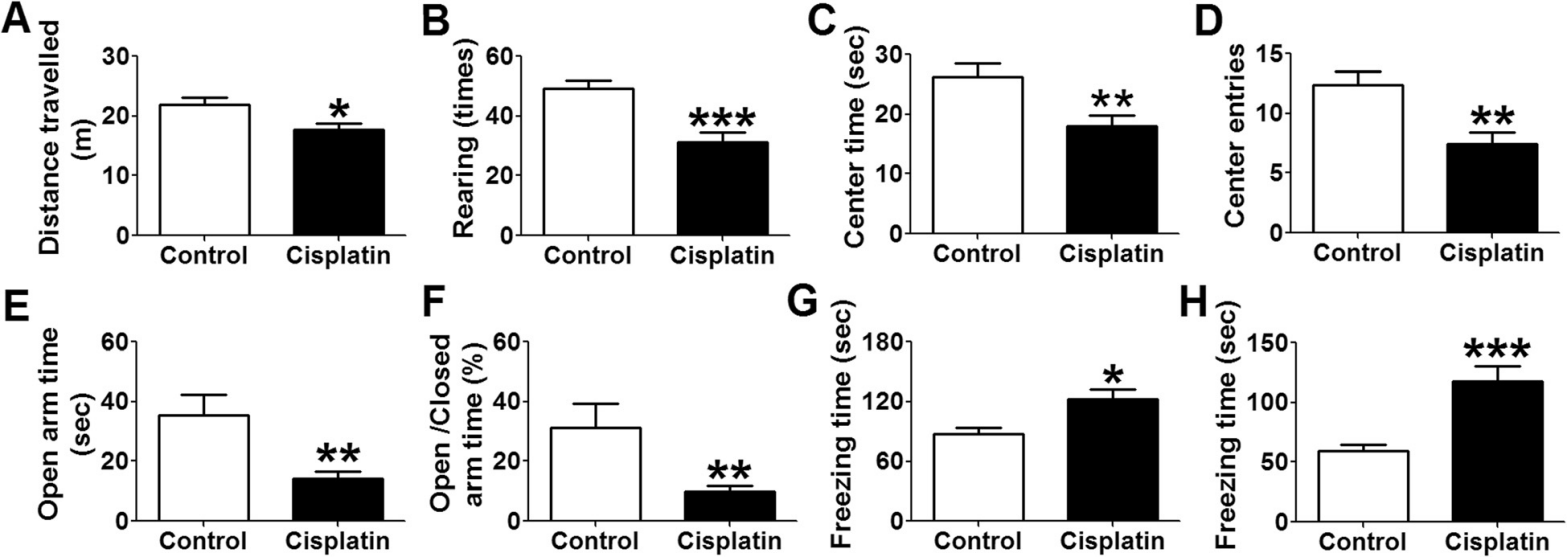
Anxiety-like behavior in cisplatin treated rats as determined in the OFT and the EPM. **a** Total horizontal distance traveled, (**b)** the number of rearings, (**c**) the time of spent in the center, (**d**) the number of entries into the center, and (**h**) freezing time in an OFT, (**e**) time spent in open arms, (**f**) percentage of time spent into open arms versus closed arms and (**g**) freezing time for the EPM. Values are given as means + SEM. n = 17 for controls and n = 19 for cisplatin-treated rats. An asterisk indicates levels of significance for the difference between controls and cisplatin-treated rats. * p < 0.05. ** p < 0.01, *** p < 0.001 vs. control rats

Cisplatin-treated rats exhibited a lower total horizontal distance traveled (horizontal activity; t = 2.54, p < 0.05), a reduced number of rearings (vertical activity; t = 4.08, p < 0.001), a reduced time spent in (t = 3.01, p < 0.01) and number of entries into the center (t = 3.29, p < 0.01) during the 5-min testing session (Fig. 1a, b, c, d). In contrast to the controls, rats with cisplatin treatment exhibited strongly increased freezing behavior (t = 4.21, p < 0.001; Fig. 1h).

### Anxiety-like behavior

To confirm the anxiety-like behavior we performed elevated plus-maze (EPM) test. The EPM is a popular behavioral test for anxiety-like behavior and is thought to result from natural aversion of rats to explore elevated and open areas [20].

The EPM tests showed that cisplatin-treated rats exhibited a reduced time in the open arms (t = 3.07, p < 0.01; Fig. 1e) and a lower percentage of time spent in the open arms versus closed arms (t = 2.79, p < 0.01; Fig. 1f) when compared with control rats, suggesting increased anxiety-like behavior induced by chronic cisplatin treatment. As previously observed in the open field test (OFT), rats with cisplatin also exhibited a higher level of freezing behavior during exposure to the elevated plus-maze (EPM) (t = 2.71, p < 0.05; Fig. 1g).

### Spatial reference recent and remote memory

To investigate the learning acquisition processes and memory ability we conducted Morris water maze task [21]. The test used was described by Kesner [22] as a test of prefrontal cortex (PFC) function. Its goal is to assess the ability of rats to learn the position of the hidden platform and to keep this information online during four consecutive trials.

**Fig. 2:**
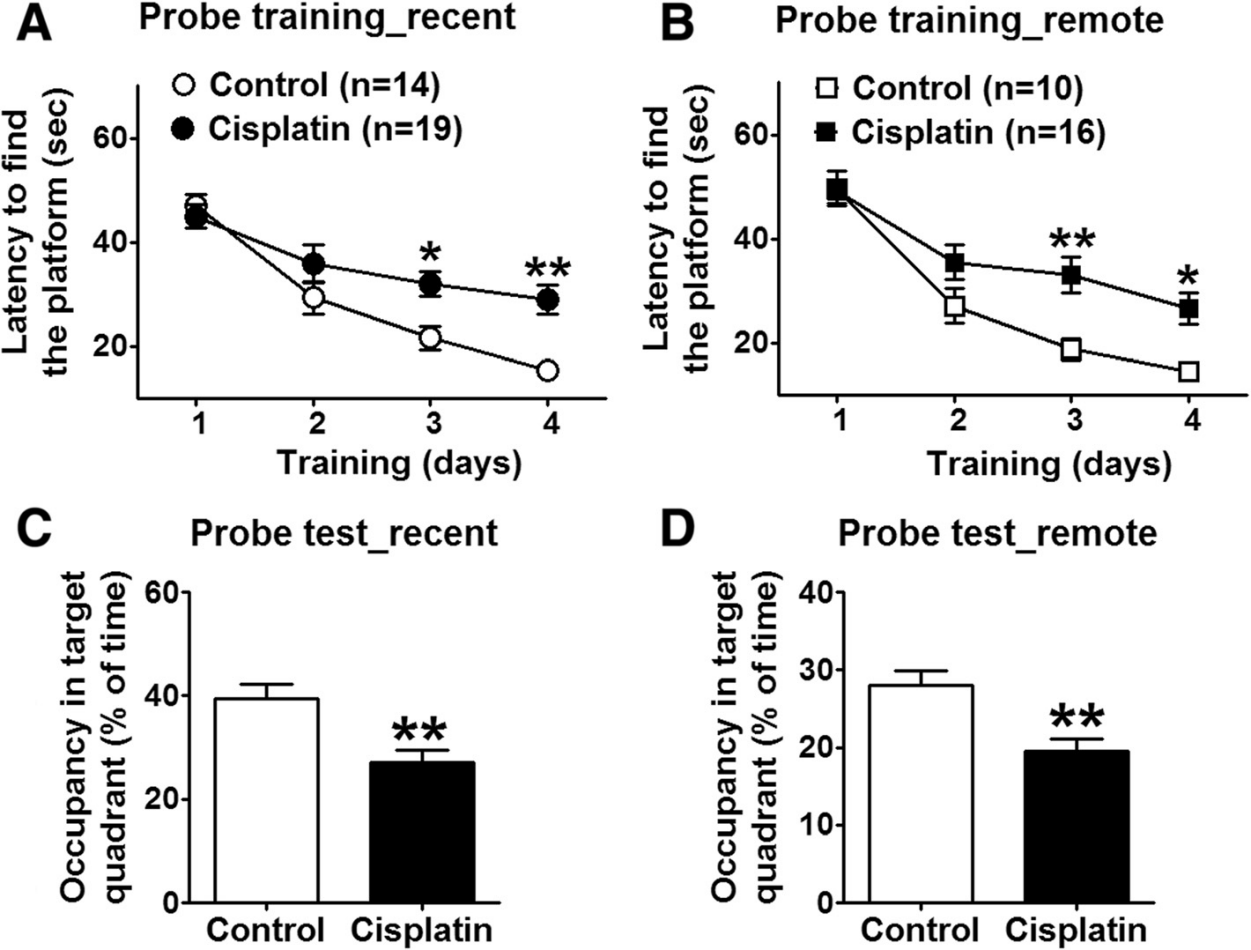
Spatial reference learning and memory impairment observed in cisplatin-treated rats exposed to the water maze test. **a - b** In the probe training sessions, the acquisition curve of cisplatin rats was impaired compared with control rats. **c - d** In 60 s probe tests, cisplatin-treated rats spent significantly lower percentage of time in the quadrant previously containing the platform than control rats, suggesting impaired memory. An asterisk indicates levels of significance for the difference between controls and cisplatin-treated rats.* p < 0.05, ** p < 0.01

Swimming speed did not change in cisplatin-treated rats during probe training (acquisition trials) in Morris water maze (MWM). Escape latencies were used to indicate the ability to learn the location of a submerged platform using visual cues. There was a significant effect on escape latencies for groups (F (1,31) = 8.03 and F (1,24) = 8.87; both p < 0.01 for recent and remote memory), for trials (F (3,31) = 35.35 and F (3,24) = 41.59; both p < 0.001) for recent and remote memory), and for their interaction (F (3,93) = 3.80 and F (3, 72) = 2.85; both p < 0.05 for recent and remote memory). These results suggest that cisplatin-treated rats spent more time trying to find the platform during 4-day acquisition over the course of several trials (Fig. 2a and b). Spatial memory was assessed in a single 60 s probe test in which rats searched the target quadrant that previously contained the escape platform. Control animals showed a significantly higher percentage of time spent in the target quadrant compared with cisplatin rats when tested in a probe trial 24-h after training (t = 3.33, p < 0.01; Fig. 2c) and in 30-day after training (t = 3.61, p < 0.01; Fig. 2d), suggesting cisplatin-disrupted memory retrieval in both the recent and remote memory retention.

### Cisplatin-induced behavioral changes in decision-making during the rat Iowa gambling task

The rat gambling task (RGT) has been developed to test the decision-making capacities in rats via a conflict between immediate and long-term gratification (food reward) [23].

**Fig. 3:**
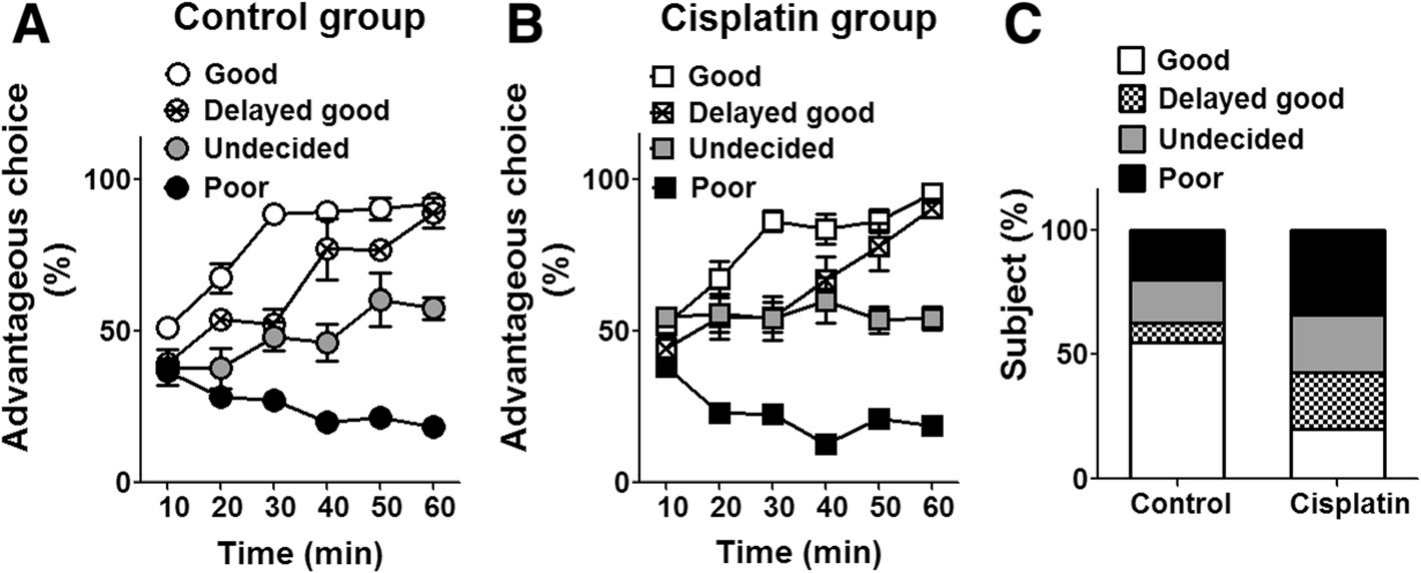
Changes in decision-making behavior induced by cisplatin using a rat Iowa gambling task. **a – b** Time course of percentage of advantageous choices for good (white), delayed good (grid), undecided (30 % gray) and poor (black) decision-makers during a 60 min RGT testing in control (A circles) and cisplatin (B squares) rats. Within the first 10 min, rats chose equally between advantageous and disadvantageous choices. They then quickly developed four distinct subgroups based on the percentage of advantageous choices during the 60 min test (>70 % preference of advantageous choices at 30 min and in the last 20 min for good decision-maker, < 70 % preference of advantageous choices at 30 min and > 70 % preference in the last 20 min for delayed good decision-maker, < 30 % in the last 20 min for poor decision-maker, and 30 - 70 % in the last 20 min for undecided maker). **c** Proportions of good performers (white bar), delayed good performers (grid bar), undecided behavior (30 % gray bar) and poor decision-making (black bar) for control group and cisplatin group. Advantageous choices (%) = numbers of nose-poke for choices (C + D) / numbers of nose-poke for choices (A + B + C + D) * 100 %. n = 24 for control group, n = 35 for cisplatin group

During the RGT different subgroups of decision-making performers were identified in both the control and cisplatin-treated rats, based on the percentage of advantageous choices (choice C and D) made during the testing session (Fig. 3a and b). During the first 10 min of the 60 min testing session, no obvious choice preference was observed in any subgroup in either the control or cisplatin group (Fig. 3a and b). Over time, some rats gradually preferred advantageous choices and finally developed good decision-making behavior (defined by advantageous choices >70 % in the last 20 min). Delayed good decision making behavior was identified when the percentage of advantageous choices in good decision-makers was below 70 % at 30 min. Poor decision-making corresponded to those with less than 30 % advantageous choices in the last 20 min. The remaining rats showed no obvious preference for either advantageous or disadvantageous choices throughout the test (between 30 % and 70 % preference for advantageous choices in the last 20 min), and were classified as undecided. The proportion of delayed-good decision-makers increased after repeated cisplatin treatments (8 out of 35 cisplatin rats, 23 %) compared to the control group (2 out of 24 control rats, 8 %), and the proportion of normal good decision makers was reduced, to 20 % in cisplatin rats compared to 55 % in controls (Fig. 3c and Table 1). The proportion of undecided performers in the cisplatin group was similar to that observed in controls (23 % vs. 20 %, cisplatin vs. control), with 34 % and 17 % poor decision-makers observed in the cisplatin and control groups respectively (Fig. 3c and Table 1). The difference in the proportions of the four subgroups between the control and cisplatin groups was significant (non-parametric Mann–Whitney test, z = −2.27, p < 0.05, Fig. 3c), suggesting that cisplatin treatment affects decision-making ability in rats

**Table 1:** Number and percentage of individuals exhibiting the behavior observed during the RGT for both the control and cisplatin groups

Number of individuals	Control group (n = 24)	Cisplatin group (n = 35)
	n (%)	n (%)
Good decision makers	13 (55 %)	7 (20 %)
Delayed-good decision-makers	2 (8 %)	8 (23 %)
Poor decision-makers	4 (17 %)	12 (34 %)
Undecided decision-makers	5 (20 %)	8 (23 %)

### Impaired LTP-like synaptic plasticity

**Fig. 4:**
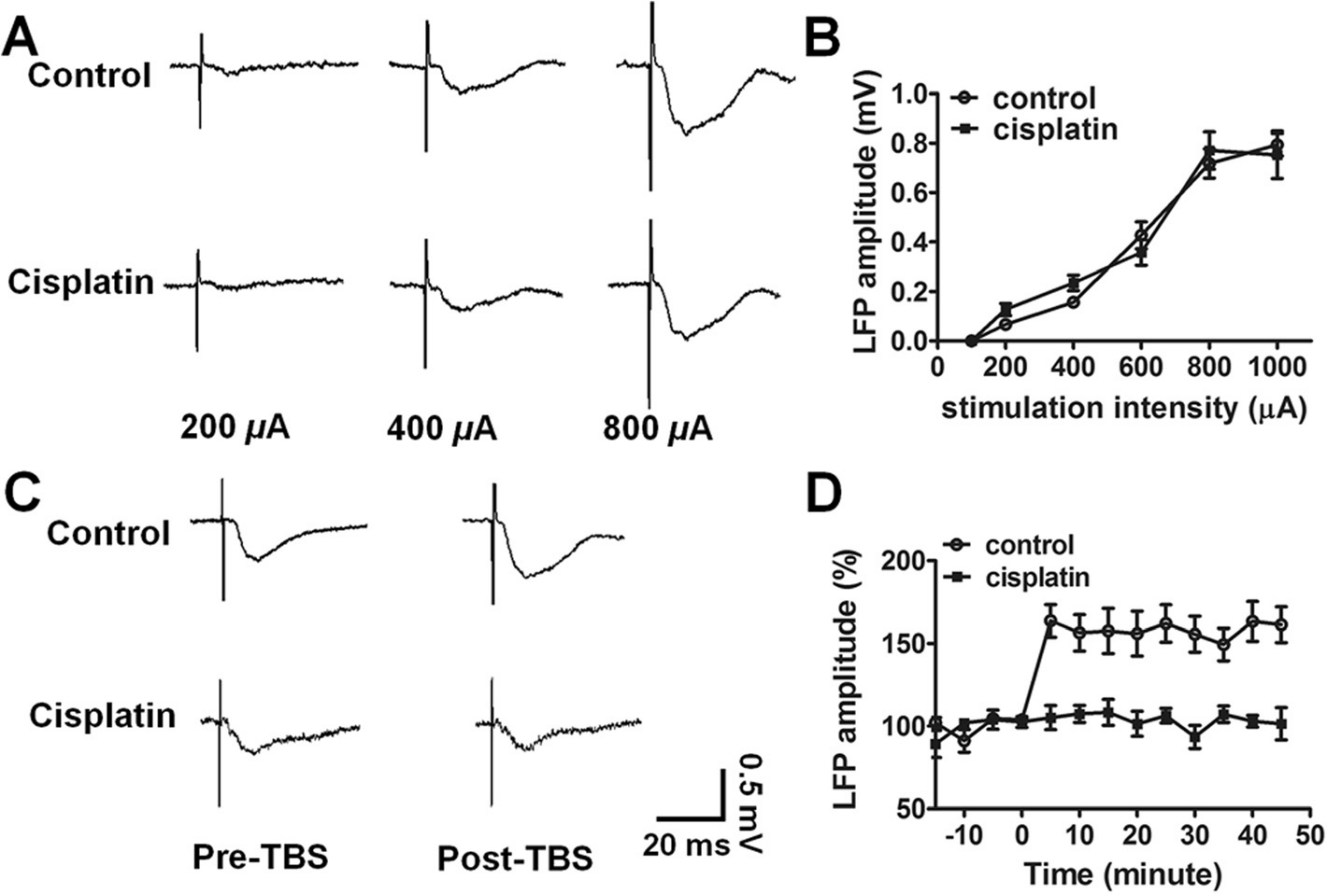
Long-term potentiation (LTP)-like plasticity in the basolateral amygdala (BLA)-ACC synapse was impaired in cisplatin-treated rats. **a** Representative ACC evoked local field potential (LFP) responses to different-intensity BLA stimuli (200, 400 and 800 *μ*A) in both control and cisplatin rats. **b** Averaged input/output (I/O) curves of BLA-ACC evoked LFP showed no significant alteration between control and cisplatin-treated rats. **c** Examples of LFP responses in the ACC to BLA stimuli before (pre-) and 40 min after (post-) theta burst stimulation (TBS) in control and cisplatin-treated rats. **d** LTP was reliably induced in control rats (n = 5). But in cisplatin-treated rats, the LTP induction was impaired when tested with a stimulate intensity that evoked about 50 % of the maximum LFP amplitude (n = 6). Results are expressed as mean ± SEM

In all rats, a range of stimuli (50 to 1000 μA) to the BLA elicited gradually increased ACC evoked LFP (Fig. 4a). No significant difference was observed between control and cisplatin rats I/O curves (F = 0.33, p > 0.05, Fig. 4b). In control rats (n = 5), after three trains of TBS delivered to the BLA, a 158.4 ± 9.6 % enhancement in LFP amplitude in response to BLA stimuli (500 μA) was observed compared to the baseline level (pre-TBS). In contrast, TBS failed to induce LTP in cisplatin-treated rats (n = 6). Representative evoked LFP are shown in Fig. 4c; there was no obvious alteration of LFP tested in pre-TBS and post-TBS condition at any time points in cisplatin rats (103.7 ± 6.4 %, Fig. 4d). These results indicate that TBS-induced LTP in the BLA-ACC synapses was impaired after cisplatin administration. We also detected impairment of LTP induction in large-scale ACC in the multiple-channel electrode study (data not shown).

### Enhanced ACC theta activities

**Fig. 5:**
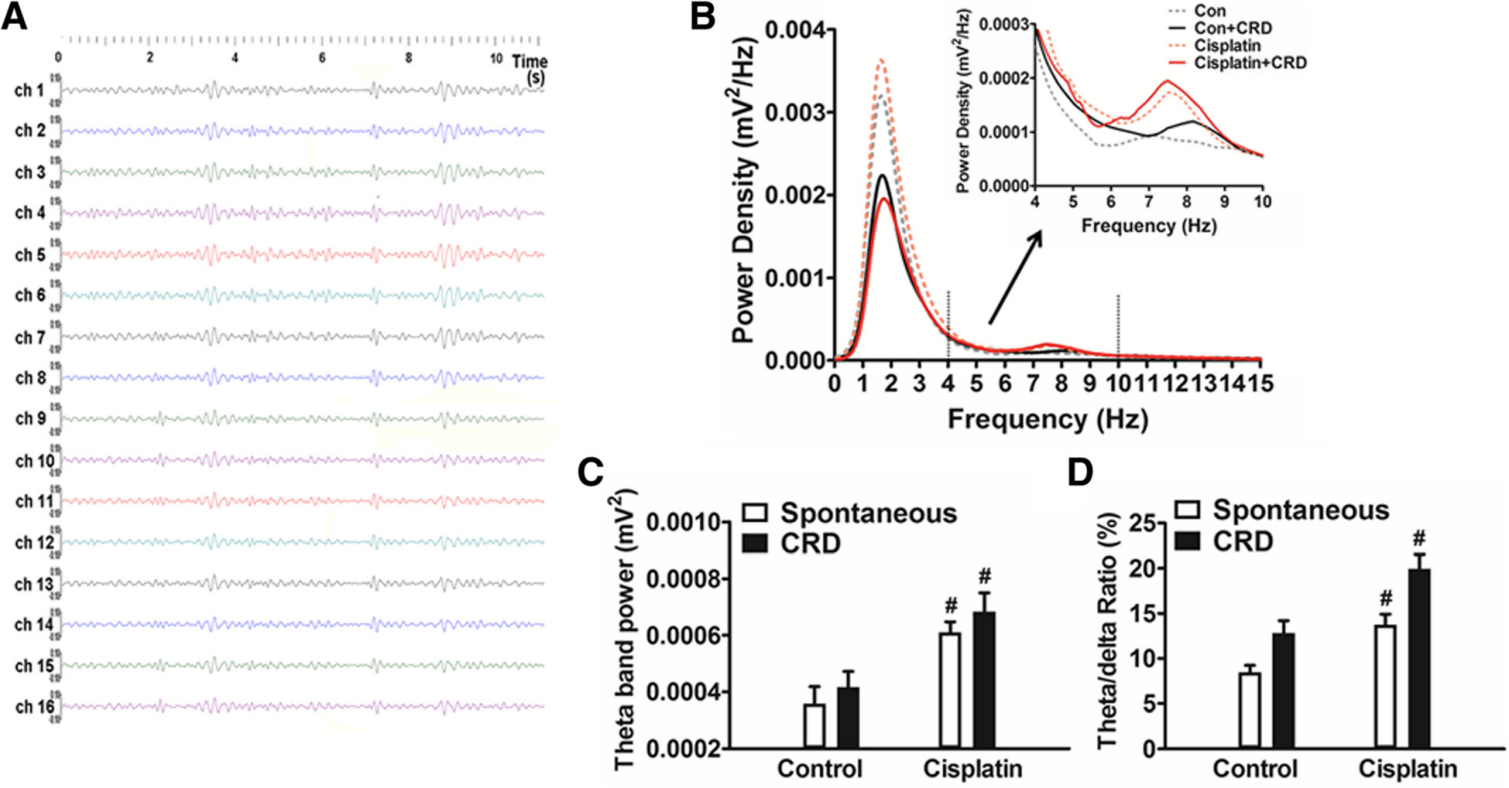
Enhanced the power of ACC theta-band oscillation (4–10 Hz) in cisplatin-treated rats. **a** The 16 channels LFP oscillations in the theta band frequencies recorded from the ACC. **b** The averaged power spectral density (PSD) showing a wider band of frequencies (0–15 Hz) in control and cisplatin-treated rats during spontaneous and CRD conditions. The main peaks of the PSD located during 1–4 Hz (delta band), the second peaks as magnified in the inset show power distributions in the 4–10 Hz (theta band) in different groups. **c** The average AUC of theta band PSD in control and cisplatin-treated rats showing increases in theta-band oscillation (4–10 Hz) power during both spontaneous and CRD stimulate conditions in cisplatin rats. **d** The histogram shows the enhancement of theta/delta ratio (theta/(theta + delta) in cisplatin rats. Data are expressed as mean ± SEM. # p < 0.05 vs. control

To characterize the theta rhythm activity of the ACC, power spectral analysis was performed during 30 s spontaneous and 30 s CRD stimulation in two groups of rats. The AUC of the theta band power are shown in Fig. 5b (n = 7 for control and 7 for cisplatin group), a significant alteration between the groups was detected (F = 7.71; p < 0.001). *Post hoc* analysis revealed that the ACC theta power during both spontaneous activity (t = 3.32; p < 0.05) and visceral pain condition (60 mmHg CRD; t = 3.28; p < 0.05) were enhanced in cisplatin rats compared with control. In control rats the ACC theta power was 3.58 ± 0.64 and 4.17 ± 0.55 × 10^−4^ mV^2^, while in cisplatin rats the ACC theta power were 6.10 ± 0.37 and 6.84 ± 0.60 × 10^−4^ mV^2^ in spontaneous conditions and during CRD stimulation respectively. Theta/delta ratios were also evaluated (Fig. 5d); *post hoc* analysis revealed that in cisplatin-treated rats under spontaneous conditions, ACC theta/delta ratio was enhanced from 8.50 ± 0.80 to 13.77 ± 1.08 % (t = 2.91; p < 0.05). And during CRD stimulation, the theta/delta ratio was increased from 12.85 ± 1.44 to 19.94 ± 1.60 % (t = 4.08; p < 0.05). These data indicate that cisplatin enhances ACC theta activities.

### Disrupted spikes phase-locking to theta oscillation in the ACC in cisplatin-treated rats

**Fig. 6:**
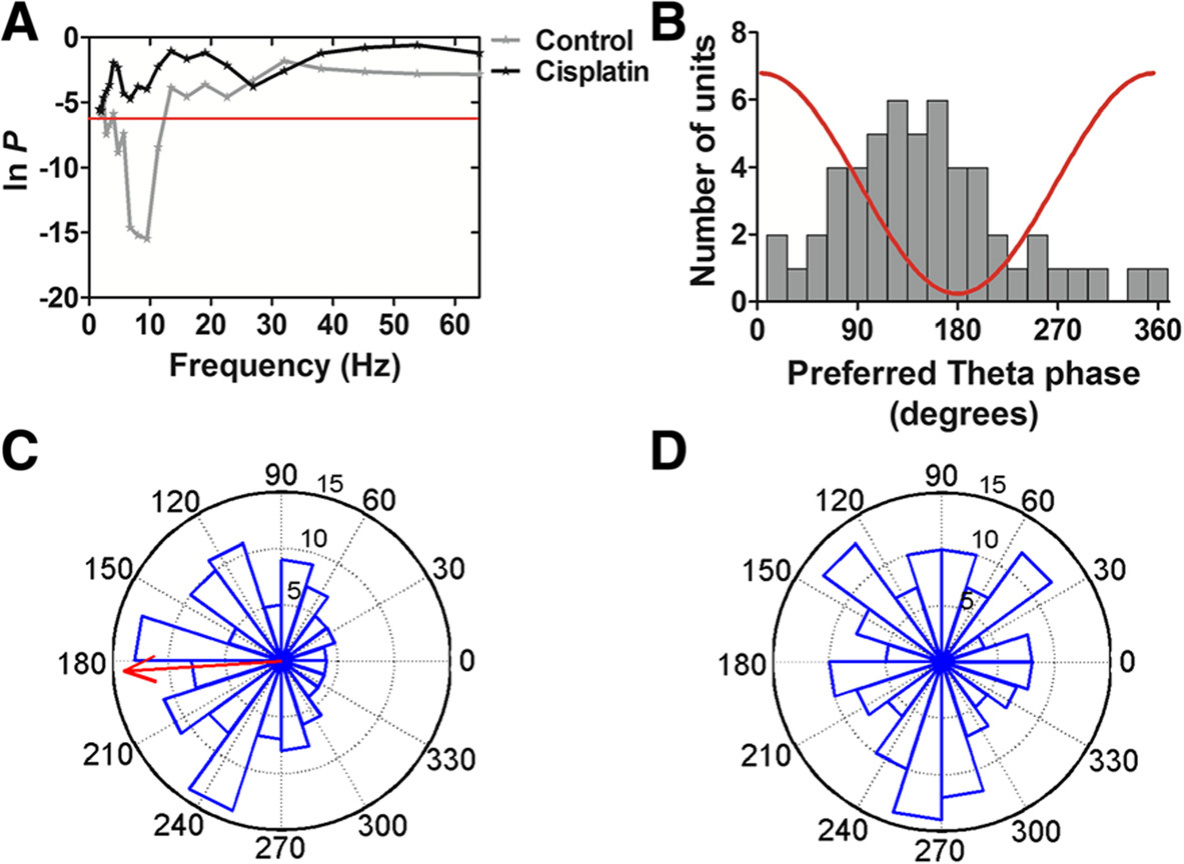
Cisplatin disrupted the spikes phase-locking in the ACC. **a** Test of significance of phase-locking as a function of frequency (1 – 64 Hz). The threshold (red line) for significant phase-locking was set to p = 0.0023 (0.05/22, Bonferroni corrected). The shown phase-locked neuron in the control rat (gray line) exhibited maximal phase-locking at 9.5 Hz while the un-phase-locked neuron in the cisplatin-treated rat (black line) showed no significant phase-locking in theta range. **b** Histogram of the preferred phase of all phase-locked neurons in the control rats (n = 53 of 120). The figure shows most phase-locked neurons preferred to fire during the descending phase or at the trough of the oscillation. The red line is a schematic of the theta cycle. **c** The polar-histogram of the spike-field phase distribution of the phase-locked neuron from the control rat shown in (A). The figure shows the majority of spikes of this neuron fired close to 180°. The mean phase shown by the red arrows indicates this neuron preferred firing at 184° of the theta oscillation. The vector length R = 0.23. **d** Polar-histogram of the spike-field phase distribution of the un-locked neuron from the cisplatin-treated rat shown in (A). The figure shows this neurons action potential firing at random angles of the theta cycle oscillations suggesting disrupted phase-locking in rats following cisplatin treatment. The vector length R = 0.03

To further investigate the strength of coupling between spike timing and LFP at any given frequency, we next asked whether spike phase distributions in the local theta oscillation of local field potential were changed in rats after cisplatin treatment. As expected, we found that in control rats 44.2 % of neurons (53 of 120 neurons) fired spikes that were phase-locked to the LFP oscillations in theta range (Fig. 6a, p < 0.0023, 0.05/22, Rayleigh’s test). The preferred frequency of phase-locked neurons was 8.03 ± 0.98 Hz. In contrast, in cisplatin group, only 12.8 % (19 of 149 neurons) showed phase-locking at theta range. In control rats the neurons had a range of phase preferences, with the majority (27 of 53 neurons, 51 %) firing close to the trough of the theta band oscillation (±45° around trough; Fig. 6b). Examples of phase distributions of a phase-locked neuron in a control rat and an un-phase-locked neuron in a cisplatin rat are presented as polar-histograms (Fig. 6c and d). The phase-locked neuron in the control rat showed most spikes firing at between 120° and 240° of the theta cycle with a mean-phase of 184°. However, in cisplatin-treated rat the un-phase-locked neuron displayed random firing. Together, the results indicate the disruption of the ACC phase distribution of single neuron spikes in relation to ongoing local field potential in cisplatin rats.

### Desynchronized theta activities in BLA and ACC

**Fig. 7:**
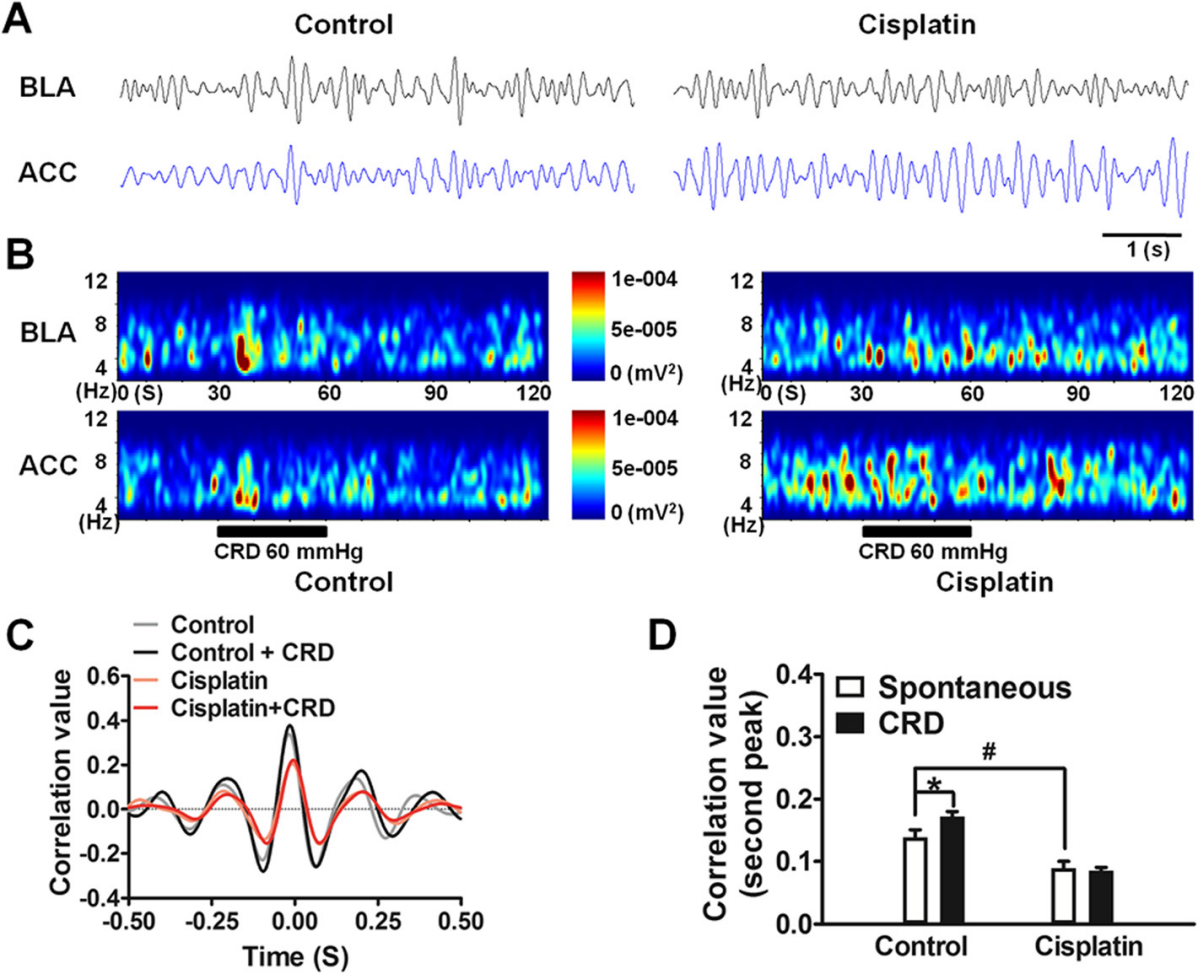
Desynchronized theta activities between the BLA and ACC in cisplatin rats. **a** Representative traces of spontaneous theta band field potential in the BLA (black line) and ACC (blue line) in control (left) and cisplatin (right) rats. **b** Time-varying power spectra of BLA (upper) and ACC (bottom) LFP_θ_s in control and cisplatin rats. **c** The averaged cross-correlograms of control and cisplatin rats for both spontaneous activity and during visceral pain stimulation (60 mmHg CRD). **d** Statistical analysis revealed that the cross-correlation value (the second positive peak) was decreased in spontaneous theta activity in cisplatin-treated rats compared with control group. Meanwhile, in control rats, CRD significantly enhanced the correlation value when compared with spontaneous activity. In cisplatin rats there was no changing during CRD stimuli compared with resting state. Data are expressed as mean ± SEM. *, # p < 0.05

To examine the synchronous activities between BLA and ACC we compared theta oscillations of the local field potential at rest and during noxious stimulation in both the control and cisplatin-treated rats. Here, the CRD-induced responses provide an index of how the system responds to activation. The original traces of spontaneous theta activity are shown in Fig. 7a. Time-varying power spectral analysis of 120 s recordings revealed that the concentrated neuronal activities in the ACC and BLA of control rats, both at rest and during visceral pain stimulation (CRD 30 s), became dispersed at theta frequencies in cisplatin rats (Fig. 7b). Cross-correlation analysis allows quantitative evaluation of the synchronized activity in the LFP. By averaging the cross-correlograms and taking the second positive peak as a quantitative measure (Fig. 7c), a significant difference was detected (F = 21.35, p < 0.001; Fig. 7d). *Post hoc* analysis revealed that in spontaneous conditions (basal) the correlation values were decreased in cisplatin rats, from 0.147 ± 0.013 in control rats (n = 6) to 0.090 ± 0.005 in cisplatin rats (n = 6; t = 3.93, p < 0.05). During visceral pain stimulation the cross-correlation values of control rats were increased from 0.147 ± 0.013 at basal values to 0.174 ± 0.010 during CRD (t = 3.42, p < 0.05). In contrast, in cisplatin rats visceral pain stimulation failed to alter the cross-correlation value between BLA and ACC (0.090 ± 0.010 of basal vs. 0.085 ± 0.005 during CRD, t = 2.77, p > 0.05). The result suggests desynchronized activity between BLA and ACC in both resting and visceral pain stimulation in cisplatin-treated rats. This indicates that the BLA and ACC may only loosely interact for dynamic information transfer after cisplatin treatment.

## Discussion

Using a model of chemotherapy associated with emotional and cognitive deficits, this is the first study to investigate the synaptic plasticity characterized by the phase-locking of the neural spike firing with the ongoing theta oscillation of the LFP. We also clarify the synchronization of large scale theta oscillation in the BLA-ACC pathway. Single cytostatic agent cisplatin was used instead of a chemotherapeutic cocktail in a clinical setting. Cognitive functions were examined up to two months post treatment and the animals were free of cancer and other diseases.

In the EPM [20], we observed that cisplatin-treated rats decreased the amount of time they spent in the open arms, and significantly increased their freezing time suggesting the development of anxiety-like behavior. Anxiety is an instinct for promoting adaptive survival by elusion of unnecessary danger. However, excessive anxiety is unfavorable reducing even the behavioral activity that is necessary for adaptation. The amygdala plays a key role in the expression of anxiety or fear, and the medial prefrontal cortex is important for regulating the amygdala-mediated expression of fear [24].

In the spatial reference memory task, the cisplatin-treated rats showed impaired learning acquisition processes. Furthermore, these rats spent less time in the platform quadrant compared to control rats in both recent and remote probe test suggesting impaired recent and remote memory.

The hippocampus plays a critical role in spatial memory formation [8, 25], however, as these memories mature, they may become more (or even exclusively) dependent on extra hippocampus structures. Previous studies demonstrated that the medial prefrontal cortex (mPFC) specifically the ACC play a key role in the expression of remote spatial memories [26, 27]. Employing a conditional place avoidance test we recently showed, that ACC neuronal activities play a key role in visceral pain aversion memory processing in awake rats [28]. Using a visceral hypersensitive rat model we have demonstrated that ACC hypersensitivity can be observed up to 7 weeks after the initiation of colonic anaphylaxis and is independent of mucosal inflammation. The prolonged ACC sensitization enhanced visceral pain responses by descending facilitate system [29], this suggests triggering of pain memory in the ACC neuronal circuitry [30–32]. More recently, electrophysiological recording showed LTP of local field potential can be reliably induced in the medial thalamus-ACC synapses by TBS condition stimulation in normal rats [33]; however, this canonical LTP induction was occluded in chronic pain state [33]. Interestingly, similar results were observed in the present studies, the data demonstrate for the first time that cisplatin effectively blocks the expression of LTP at amygdala-ACC synapses in vivo. This occlusion approach has been commonly used to investigate whether the plasticity obtained *in vitro* by electrical stimulation is a reliable model for studying the mechanisms of learning and memory [34]. These results suggest that chemotherapy agents could causally interrupt learning and memory [8]. In line with the present results, a similar LTP occlusion has been found after a fear paradigm in amygdala [35] and hippocampus [36].

In the brain, there are both small- and large-scale levels of organization [37]. Neural oscillations play a basic role in coordinated activity during normal functional brain processes [38, 39]. Several lines of evidence suggest that the theta band (4–10 Hz) in the neural network, which temporally links single neurons into assemblies, cooperatively facilitates synaptic plasticity [40]. Our spectral analysis of ACC local field activities revealed that on average, the cisplatin-treated rats exhibited higher spectral power over the frequency range in theta band (4–10 Hz). However, the frequency at which theta power reached maximum was not significantly shifted. It appears that cisplatin affects the physiological expression of theta oscillation activity, but does not alter the precise frequency of this oscillation. This abnormal theta oscillation power is consistent with previous observations that neurotoxicity of Ecstasy causes an increase in theta power in EEG which is correlated with a decline in attention and memory [41]. An animal study also showed spatial memory deficit accompanied with hippocampal theta power enhancement caused by neurotoxins [42].

A growing body of research evidence suggests that neurons transmit information not only in terms of their firing rates but also by varying the timing of the spikes corresponding to neuronal oscillations [37]. Induction of LTP is favorable when higher frequency electrical stimulations were arrived on the positive phase of theta oscillations [43] Furthermore, behavioral studies in humans and animals have consistently shown that [9] brain oscillations coordinate with timing of single-neuron activity [17], synchronously discharging cells are more effective at driving neurons at subsequent processing stages [17, 44, 45] than uncoordinatedly responding cells. In addition, to advance the understanding of the timing relationship between spikes and ongoing theta oscillation, it is critical to investigate the angular distributions of spikes with the theta oscillation, and clarify the significance of phase-locking of spikes in theta oscillation [17]. We found that in control rats 44.2 % of ACC neurons fired spikes that were phase-locked to the LFP oscillations in the theta range. The preferred frequency of phase-locked neurons was 8.03 Hz, with maximal activity during the descending phase and at the trough of the oscillations. In contrast, only 12.8 % ACC neurons showed phase-locking at theta range in the cisplatin group. Together, these results suggest that chemotherapy disrupts phase distribution of ACC neuron spikes in theta oscillation of LFP.

Decision-making is the result of the integration of several executive brain areas and has emerged as a crucial theme in neurophysiological studies of cognition. Animals are individuals that can exhibit human-like cognitive characteristics, such as the ability to acquire and reason with causal knowledge [46, 47]. Decision making is complex ; it can be impaired in psychiatric disorders, such as attention-deficit/hyperactivity disorder. The Iowa gambling task (IGT) is frequently used to assess decision-making performance in a clinical setting [48]. Our RGT results show that good, delayed-good, undecided, and poor decision-makers could be identified in the RGT [49]. In the cisplatin-treated group we observed decreases in the percentage of good decision-makers, and increases in the percentage of maladaptive behavior (delayed-good decision-makers plus poor decision-makers). Investigators have proved that the ACC plays a crucial role in selecting proper actions when faced with different benefits in an unsure environment [50] through signaling error-likelihood [51]. The ACC also plays a key role in choosing appropriate actions when the environment is dynamic [52, 53] related to the information about the costs and benefits [54]. It appears that the dysfunction of the phase-locking of the neural firing with the theta oscillation may contribute to mediate the decision-making deficits following cancer chemotherapy [49]. In addition, and also in agreement with other reports [55], our results revealed that high levels of anxiety were associated with poor decision-making using the RGT reward model. This is also in line with human data that highly anxious subjects performed worse on the IGT [56].

Advantageous decision-making in the gambling task also requires an intact amygdala [57], which is a key player in emotions and affective disorders. Accumulating evidence implicates phase synchronization and large-scale integration of neuronal activities in different regions as a mechanism linking functionally related regions of the brain [37, 58]. The direct and indirect connections between ACC and amygdala have been well determined anatomically [11, 13]. Electrical stimulation of the BLA has been shown to change neuronal firing in the mPFC, with latencies consistent with monosynaptic and polysynaptic pathways [59]. Behavioral studies have also shown that the BLA-ACC synaptic pathway participated in various cognitive functions. For example, in chronic pain state, the cognitive impairment was caused by amygdala-driven prefrontal cortical deactivation [60]. Synchronous oscillatory activity in the theta band has been suggested to mediate information flow between functionally related brain regions during learning and memory retrieval [61]. Previous studies have shown that the synchronized activity between frontal and hippocampal regions was modulated by anxiety [62], and enhanced during the execution of working memory tasks using human EEG recording [63]. In the current study, we tested our hypotheses that cisplatin leads to desynchronization in the amygdala and ACC correlated with deficits in learning and memory. By simultaneously recording local field potentials in the ACC and the BLA, cross-correlation and time-varying power spectral analysis of the theta oscillations at rest revealed a pattern of dispersion of theta band activity and reduction in correlation values of cisplatin rats when compared to the control rats. Furthermore, noxious stimulation (CRD) enhanced the synchronized theta activities between BLA and ACC in normal rats but in contrast, non-synchronized theta activities were observed during CRD after cisplatin administration. These data might be particularly relevant to the failure of introduction of LTP in the BLA-ACC pathway after administration of cisplatin. It has been well demonstrated that LTP is induced preferentially on the positive phase of theta oscillations. In cellular studies it was clearer that whether a given stimulus triggers synaptic plasticity can depend on when the stimulus arrives relative to the phase of ongoing theta oscillation [43]. It appears that higher frequency of theta burst stimulation (TBS)-induced LTP shared similar modality with the synchrony of theta oscillation of LFP in the BLA-ACC pathway. We speculate that the theta synchronized activity between circuits might promote the induction of LTP by electrical higher frequency TBS in these circuits. Together these results provide the first clear indication that chemotherapy disrupts the phase-locking of single neural firings with respect to the ongoing theta oscillation, and further interrupts the synchrony in the BLA-ACC pathway, which correlates with blocking LTP induction in this pathway. These observations directly complement recent seminal findings showing that in human studies [17], the interruption of neuronal synchrony appears to impact cognition and behavior.

A limitation of the present study is that electrophysiological studies were performed under anesthesia. We observed an increase in theta/delta ratios after cisplatin treatment. However, another report showed that temozolomide, another drug for chemotherapy, reduced theta/delta ratio in hippocampus of conscious rats after 4 weeks of treatment associated with the disruption of learning [61]. A series of experiments have shown that theta oscillations are quite different among anesthetized and different sleep-wake states in awake rats. Kramis *et al.* reported two types of theta rhythm in hippocampus, one is sensitive to astropine and the other is abolished selectively during ether or urethane induced anesthesia [64]. More recently, other researchers indicated that under anesthesia, hippocampal activity switched from large-amplitude, irregular activity to a theta state, and the theta/slow oscillation ratio increased when transiting anesthetized to the waking, which was mediated by cholinergic [65] and serotonergic neurons [66]. How the neural network between the ACC and amygdala is coordinately activated in the spatial memory and RGT reward models in awaken state remains unexplored. Future studies are clearly needed, using chronic implants of arrays of multi-electrodes and recording from neuronal ensembles during animal behavior. This will enable exploration of the neural mechanisms underlying the functional connectivity between the ACC and amygdala during events involving learning, memory retrieval, and decision-making.

In conclusion these results provide the first direct evidence that cancer chemotherapy can reduce the coherence and interrupt the phase-locking of ACC neuronal spikes with the ongoing theta band of the LFP. More importantly, the disruption of the synchronized theta oscillations and impairment of induction of long-term potentiation of the BLA-ACC pathway are associated with emotional and cognitive deficits in rats following cancer chemotherapy.

## Materials and methods

### Animals

A total of 59 male Sprague–Dawley rats were used in these experiments; they were housed in standard laboratory conditions (temperature at 23 ± 1 °C and 12/12 h light–dark cycles with lights on at 7:30 a.m.) The rats were allowed to adapt to their environments for at least 5–7 days before the first injection of cisplatin and were handled for 3–5 min daily for 2–3 days before beginning the behavioral experiments. Rats had free access to water, but had moderate food deprivation during the decision-making testing. All surgical and experimental procedures were conducted according to the guidelines laid down by the NIH in the US regarding the care and use of animals for experimental procedures and were approved by the Committee on Use and Care of Animals at City University of Hong Kong and the licensing authority to conduct experiment from Department of Health of Hong Kong (No. 13–89 in DH/HA&P/8/2/5 Pt.2).

### Drug treatments

The cisplatin dosage and dosing schedule used in the present study were based on the clinical usage of cisplatin treatment into the abdominal cavity in cancer patients, slightly modified according to previous description in rodents [4]. Cisplatin (International Laboratory, USA) was dissolved in 0.9 % NaCl (0.4 mg/ml), warmed to 45 °C and injected with 1.0 ml/100 g rat. The rats weighing 250–300 g at the beginning of the experiment received intraperitoneal injections of cisplatin or 0.9 % NaCl once every 7–8 days for a total of 6 times in recent memory testing group and 8 time for remote memory group (Table 2).

**Table 2:** Experimental design

Recent memory		Remote memory	
Experimental day	Treatments	Experimental day	Treatments
1, 2…13, 14	Drug injections	1, 2…13, 14	Drug injections
15, 16	Habituation	15, 16	Habituation
17	OPT, EPM	17	OPT, EPM
18, 19, 20, 21, 22	Recovery phase before MWM	18, 19, 20, 21, 22	Recovery phase before MWM
23, 24, 25, 26	MWM training	23, 24, 25, 26	MWM training
27	MWM recent testing	27, 28…55, 56	Drug injections
28, 29, 30, 31, 32	Recovery phase after MWM	57	MWM remote testing
33, 34, 35	Food restriction for RGT		
36, 37, … 40, 41	RGT training		
42, 43	RGT testing and Electrophysiological recording		

Cisplatin or saline was administrated on days 1, 7, 14, 21, 28, 35 for recent memory group and days 1, 7, 14, 21, 28, 35, 42, 49 for remote memory group

### Behavioral assessments

Behavioral assessments began after 3 cisplatin administrations. The animals were tested sequentially in a series of behavioral tests in the following order: (1) open field test (OFT), (2) elevated plus-maze (EPM), (3) Morris water maze (MWM), (4) rat Iowa gambling task (RGT). The rats were given a recovery phase of 5 days before, and 5 days after MWM to maintain a fair general condition during chronic cisplatin treatment. All behavioral procedures were conducted during the light phase of the day. To minimize possible circadian influences on the rats, cisplatin and control rats were observed alternate basis.

#### Open field test (OFT)

The OFT is often used to assess exploration in a novel environment and offers a preliminary screen for anxiety-related behavior in rats [19]. Before the actual testing, animals were habituated to the behavioral testing room for 1–2 h sessions on 2 consecutive days. During a 5-min testing, rats were placed individually in the center of a 40 × 40 cm square drawn in the middle of a black square arena (80 × 80 × 40 cm) and then allowed to explore the field freely. The apparatus was cleaned between rats using 70 % ethanol. Spontaneous exploration behavior in the OFT were observed and recorded by ANY-maze (Stoelting Co., Wood Dale, IL, US), as indicated by total horizontal distance traveled (m), number of rearings (times), time spent (s) and number of entries (times) in the center and peripheral areas, and freezing time (s).

#### Elevated plus-maze (EPM)

The EPM is a popular behavioral test for anxiety-like behavior and is thought to result from natural aversion of rats to explore elevated and open areas [20]. The apparatus was made of brown acrylic plastic with two sets of opposing arms (open arms: 50 × 10 cm; closed arms: 50 × 10 × 40 cm), 50 cm from the ground. Rats were placed individually in an open field for 5 min and then allowed to start exploring the maze freely from the junction of the two sets of arms (10 × 10 cm) facing one open arm in a 5-min test. The maze was cleaned between rats using 70 % ethanol. Time spent in each arm was recorded using the ANY-maze with entries being defined as 85 % of the area of the animal being present in the area entered. Time spent in open arms, especially the percentage of open arm time versus closed arm time was evaluated to assess anxiety.

#### Morris water maze (MWM)

The MWM was used to assess and compare spatial learning and memory in cisplatin-treated and control rats as described previously [26, 67]. Experiments were performed in a black circular tank (a diameter of 150 cm with 60 cm depth), which was filled with the opaque water (22–24 °C) by adding black nontoxic paint. A circular platform (diameter of 10 cm and 25 cm high) submerged 2.0 cm below water level in the center of one of the four quadrants (target quadrant). Behavioral data of the animal in the water maze were acquired using the ANY-maze by a digital video camera located above the center of the tank.

Four consecutive days of probe trainings were performed to assess special learning. Rats were released from 4 randomized release points facing the tank wall to learn to locate a hidden platform in 60 s using visual orientation cues on the walls of the tank and on the curtains around the tank. Rats either found the platform by themselves or were guided by the experimenter, and remained on the platform for 10 s. Four trials were conducted each day with intervals of 30 min between each. Twenty-four hours or thirty days after the last training session, a single probe test was performed to assess recent or remote spatial memory, respectively. The hidden platform was removed to assess memory retention for the submerged platform location and rats were released from the farthest position of original platform and allowed to search the target quadrant that previously contained the escape platform. Swim paths for all trails were recorded and latency to reach platform (sec), distance traveled (m), swimming speed (m/s), and the amount of time spent in the target quadrant (% of total time) were calculated to assess spatial recent or remote memory acquisition and retention/retrieval.

#### Rat Iowa gambling task (RGT)

The rat Iowa gambling task was used to detect decision-making. The rats made choices from options associated with different amounts of reward (food pellets) but also different amounts and likelihoods of penalties (time-outs) [18]. The training and test procedures for the rat were identical to the ones previously described by Rivalan et al. [49].

The task was performed in four polyvalent conditioning boxes (28 × 30 × 34 cm) adapted from five-choice serial reaction time chambers (Imetronic, Pessac, France). In each box, there were 4 nose-poke holes illuminated with white LED on the front curved wall, a food dispenser providing food pellets (45 mg, TestDiet, USA) at the back wall and a transparent central opening partition (7 × 7 cm) dividing the box into two chambers at the middle. Infra-red detectors were equipped in holes to detect the nose poke and connected to the food dispenser.

Prior to the actual training, food was restricted over a 3-day period following a 1-day fasting period. Simultaneously, 50 reward pellets per rat were put inside the cage every day to make sure the rats became habituated to their taste. During the training phase, daily food was restricted for each rat to about 5 g of food per 100 g body weight and rats usually took 5–7 days to make the association between nose-pokes in illuminated holes and food rewards in the food dispenser. In order to guarantee that the selection of the nose poke was a conscious choice, the rats were trained to associate a single nose-poke with one food pellet delivery according to a criterion of at least 100 pellets obtained within a 30-min session, followed by two consecutive nose-pokes with one food pellet delivery with the same criterion. Two final 5-min training sessions were conducted to habituate the rats with the quantity of pellets that could be obtained during the test. The first session was set by two nose-pokes with two pellets at a time (maximum 30 pellets) after making a choice and the other with one pellet (maximum 15 pellets).

The test procedure was performed the following day and lasted 60 min or was cut off by 250 pellets obtainment. Rats were free to make choices among the four holes (A-D) as during the training phase; however, different choices were associated with different outcomes set as follows: choices A or B related to two pellets each time as immediate reward, but had separately 1/2 probability to trigger a long penalty time-out (222 s) or 1/4 probability for a very long penalty time-out (444 s), during which period no pellet can be obtained; choices C or D associated to smaller immediate reward (one pellets each time), but also smaller penalty (1/4 chance for 12 s time-out, or 1/2 chance for 6 s time-out). Although the immediate reward of choice A and B was twofold that of choice C and D, in the long run the choice C or D would allow for the rats to obtain more food pellets compared to choice A or B. Thus the choices C and D were advantageous choices, while the choices A and B were disadvantageous choices. The percentage of advantageous choices (number of nose-pokes (C + D) / number of nose-pokes (A + B + C + D) * 100 %) was used as a criterion to distinguish the good (>70 % preference of advantageous choices during the last 20 min of RGT test), undecided (30 % - 70 % preference) and poor (<30 % preference) decision-makers. Among the good decision makers, delayed-good decision-making was determined when the percentage of advantageous choices was below 70 % at 30 min and above 70 % in the last 20 min.

### Electrophysiological recordings

The electrophysiological recordings in both the control and cisplatin-treated rats were performed after the series of behavioral tests.

#### Evoked local field potential (LFP) recording

LFP in the anterior cingulate cortex (ACC) elicited by the electrical stimulation of the basolateral amygdala (BLA) was used as a quantitative measure of synaptic potency in BLA-ACC pathway. Eleven male Sprague–Dawley rats (5 control and 6 cisplatin-treated rats) were anesthetized (with 1.5 g/kg urethane, i.p.) and placed in a stereotaxic frame with body temperature maintained at 36.5 ± 0.5 °C. A glass recording microelectrode filled with 2.0 M NaCl was slowly lowered into the ACC (AP 2.0 - 3.8 mm, ML 0.5 - 1.0 mm, depth 1.5 - 3.5 mm). A bipolar tungsten electrode was placed in the ipsilateral BLA (AP −3.0 to −3.3 mm, ML 4.8 - 5.3 mm, depth 6.7 - 7.5 mm). To evaluate synaptic potency test stimuli (500 μA, square wave pulse, duration: 0.2 ms) were delivered at 0.033 Hz. After responses stabilized, variations in the stimulus current (from 50–1000 μA, increasing 50 μA intervals) were delivered in order to generate input–output curves (I/O). The details of the procedure were similar to those of previous publications [33, 68].

#### Long-term potentiation (LTP) induction

To examine the synaptic plasticity, LTP was induced by applying theta burst stimulation (TBS, three sets of 10 trains with 10 s intervals; each train consisted of 10 bursts at 5 Hz containing 5 pulses at 100 Hz) to the BLA. The test stimulate intensity was chosen to evoke 50 % of the maximum amplitude of evoked LFP. Details of the procedure have been described previously [33].

#### Multiple electrodes recording

In brief, rats were anesthetized and placed in a stereotaxic frame. Two small holes (1–2 mm wide) were drilled above the BLA and ACC to insert 16-channel silicon-based electrodes (Plexon, Dallas, TX). The silver grounding wires from the electrodes were wrapped around the mounting screws. Electrodes were slowly advanced using a micropositioner until clear neuronal action potentials in most recording channels were observed on-line (OmniPlex^®^ D system, Plexon, Dallas, TX). LFPs and spike firings were recorded with a 64-channel electrophysiological data acquisition system. LFPs were amplified (×20,000), band-pass filtered (0.05 - 200 Hz, 4-pole Bessel) and sampled at 1 kHz. Spikes were filtered (0.3 - 5 kHz, 4-pole Bessel) and sampled at 40 kHz. Signals from the BLA and ACC were recorded simultaneously during spontaneous activity and visceral stimulation (colorectal distention, CRD, 60 mmHg) [33].

### Histological identification

After completion of the electrophysiological recordings, a DC current (100–500 μA) was passed between the recording electrodes and ground to lesion the brain. The rats were perfused with saline followed by 4 % paraformaldehyde and then the brains were sliced at 50 μm and stained with Cresyl violet. Drawings were made of sections showing electrode tracks related to the structure of the ACC and BLA. A standard rat atlas was used as reference for reconstruction of the stimulating and recording sites [69].

### Data analysis

#### Statistical analyses

Data from the behavioral tests were analyzed by ANY-maze, POLY File (Imetronic, Pessac, France), SPSS (IBM SPSS Statistics 20) and GraphPad (Prism 5.0). Data were compared using student’s unpaired t-test and one-way and two-way analysis of variance (ANOVA) followed by the Bonferroni post hoc test. Comparisons of proportions of individuals in the gambling task were conducted using the non-parametric Mann–Whitney test. The neural data were processed off-line using NeuroExplorer 5 (Plexon, Dallas, TX) and exported to custom written MATLAB (MathWorks, Natick, MA) programs for additional analysis. Data sets were compared by one-way repeated-measures ANOVA followed by multiple comparisons of Bonferroni’s test using Prism 5.0 (GraphPad, La Jolla, CA). Results were expressed as mean ± SE. P < 0.05 was considered statistically significant.

#### Spike sorting

The single-unit spike sorting was performed with Off-Line Spike Sorter V3 software (OFSS, Plexon Inc., Dallas, TX) using combined manual and automatic sorting techniques. Spikes were identified when 3 SDs higher than the noise amplitude. All waveforms recorded from each channel were isolated as distinct clusters in 3D space based on the characteristics of spike waveforms using principal component analysis (PCA). Automatic techniques were used to generate separation of waveforms into individual clusters. Manual checking was performed to ensure that the spike waveforms were consistent and cluster boundaries were clearly separated. All isolated single-units exhibited recognizable refractory periods (≥1 ms) in the inter-spike interval (ISI) histograms [70].

#### Spectral analysis

Oscillatory brain activity in the theta (4–10 Hz) frequency range is believed to temporally linked single neuron into assemblies cooperatively facilitate synaptic plasticity [40], and plays a role in many different cognitive functions, including memory and decision making [45]. To clarify alterations in the theta power spectra following cisplatin treatment, we calculated the ACC theta power, peak frequency of the theta oscillations, as well as the theta ratio (theta/(theta + delta). In order to achieve this, the raw ACC LFPs were filtered between 1 and 100 Hz using non-causal zero-phase-shift filter (fourth-order Butterworth). The power spectral densities (PSD) were characterized using multi-taper estimates with 7 tapers and 2^13^ frequency bins (NeuroExplorer 5, Plexon, Dallas, TX). The overlapping percentage of each window is 50 %. The power spectra were normalized so that the sum of all the spectrum values equals the mean squared value of the signal. The PSD curve was smoothed with a Gaussian filter (15 bins running average), with the band power being defined as the area under the curve (AUC) of the corresponding frequency domain. The power from each animal was averaged over the 16 channels and to compute the theta/delta ratio, the AUC of the theta band power to the theta plus delta band (1–10 Hz) was calculated. To expose the time-frequency features of the theta oscillations in both BLA and ACC, the time-varying power spectra were calculated by FFT with Hann window function using NeuroExplorer 5. The spectrum units were normalized by raw PSD, so that the sum of all the spectrum values equals to the mean squared value of the signal.

#### The phase-locking of single neuron activity to the theta oscillations of local field potential

To investigate the angular distributions of spikes in relation to the theta oscillation, and clarify the significance of phase-locking of spikes with the theta oscillation, the phase distribution and Rayleigh test were performed using in house MATLAB scripts modified based on the study in [17]. To ensure the validity of the statistical results, only neurons that had at least 50 spikes were used for phase-locking estimation. Before performing the analysis, 22 different frequencies ranging from 1.6 to 64 Hz were selected, such that, f = 2^x^ with x = {6/8, 8/8, 10/8, 12/8, …, 48/8}. The LFP was then convolved with a series of Morlet wavelets centered about each of the selected frequencies and each with a length of 4 cycles. The result of these wavelet transforms, is a matrix of vectors whose absolute values (or length) and arguments (or angles) represent the amplitude and phase, respectively, of the LFP at frequency *f* and time *t*. At each of the selected frequencies, the phase angle of each spike was estimated from the inverse tangent of the real and imaginary components of the wavelet transform. The circular mean of the spike phases was calculated by taking the weighted sum of the cosine and sine of the angles. The mean angle was then the angle of the results and the mean vector length (R) the absolute value over the number of spikes. To check for phase-locking, the Rayleigh test was used to compare against uniformity by calculating the test statistic and a *p* value. A neuron was considered phase locked in theta range if the *P* value is below the threshold of 0.0023 which is 0.05 Bonferroni corrected for multiple comparisons (0.05/22, twenty-two frequencies were tested) [17].

#### Synchronized theta oscillations between BLA and ACC by cross-correlation analyses

The synchronized theta activities between the BLA LFP_θ_ and ACC LFP_θ_ were evaluated by cross-correlograms using NeuroExplorer 5 software. We aligned the 2 LFP_θ_s and chose the BLA LFP_θ_ as reference. The Pearson correlation values were calculated with a lag time ranging from −0.5 to 0.5 s with small bins (2 ms). The cross-correlation curves were smoothed with a Gaussian filter (5 bins running average). We averaged the cross-correlograms from valid electrode channels in the BLA and ACC and took the second positive peak as a quantitative measure because it locates at about 0.2 s lag time which represents theta activity at about 5 Hz [71].

## Abbreviations

ACC: Anterior cingulate cortex
BLA: Basolateral amygdala
CRD: Colorectal distension
EPM: Elevated plus-maze
LFP: Local field potential
LTP: Long-term potentiation
MWM: Morris water maze
OFT: Open field test
RGT: Rat Iowa gambling task
TBS: Theta burst stimulation
